# Orbital fistula tract - a case report of a rare cause of impossible facemask ventilation

**DOI:** 10.1186/s12871-025-02989-0

**Published:** 2025-03-12

**Authors:** Christian Volberg, Kuo-Min Kwee, David Sun, Hinnerk Wulf

**Affiliations:** Department of Anesthesiology & Intensive Care Medicine, Faculty of Medicine, Philipps University of Marburg, Baldingerstraße, 35043 Marburg, Germany

**Keywords:** Airway management, Difficult airway, Communication, Examination, Patient safety

## Abstract

**Background:**

Adequate facemask ventilation during induction of anaesthesia is a key aspect of patient safety. Difficulties can therefore be life-threatening for the patient.

**Case presentation:**

The case presented here illustrates a rare cause of an orbital fistula that led to a serious problem during facemask ventilation and demonstrates why team communication is so important.

**Conclusions:**

Preparatory errors in patient assessment and anaesthetic preparation were identified as sources of error.

## Case presentation

At the end of April 2024, a 73-year-old male patient presented to the preoperative anaesthesia consultation unit for ENT surgery of the left frontal sinus. According to the surgical registration form, the patient was scheduled to undergo left frontal sinus repair with possible opening of the intersinus septum and possible septoplasty with navigation under general anaesthesia. The patient did not state any pre-existing conditions in the self-disclosure form except for heavy snoring. According to the self-report, he did not take any medication regularly, had no allergies, and had tolerated previous anaesthesia for dental surgery well. At the time of consultation, the patient was in good general health (height 1.88 m, weight 98 kg) and physically active. The patient wore a full upper denture and the Mallampati score was recorded (Class I). Assessment of mouth opening and reclination revealed no pathological findings. Based on the unremarkable history, the ASA status classification was determined to be I. The patient gave informed consent for general anaesthesia with orotracheal intubation. At this point, there was no need for the anaesthetist to make any further enquiries about the patient’s medical history, as the patient did not provide any relevant information in the self-disclosure, apart from a previous dental operation.

The surgery was postponed until the end of June 2024. On arrival in the operating room, the patient wore an eye patch over the left eye. After a brief update with the anaesthesiologist, the WHO surgical safety checklist was processed and the patient received an indwelling venous cannula (20G) on the back of the left hand. After achieving an oxygen saturation (SpO_2_) of 100% following adequate preoxygenation according to German guidelines (10 l/min 100% oxygen for 3–4 min via face mask [1]), anaesthesia was induced with 0.3 mg fentanyl, 180 mg propofol, and 50 mg rocuronium. According to the German guideline it is not necessary to consider the possibility of mask ventilation before administering neuromuscular blockade for endotracheal intubation if there are no signs of a difficult airway [1]. After the onset of apnea and loss of eyelid reflex, facemask ventilation was initiated, resulting in an immediate loss of pressure in the anaesthesia reservoir bag. The adjustable pressure-limiting valve (APL valve) was adjusted from 15 mbar to 40 mbar and the anaesthesiology resident called the senior anaesthesiology consultant for support. The highly experienced senior physician, with over 40 years of professional experience, took over while optimizing head position and performing the head tilt-chin lift and jaw-thrust maneuvers. Despite maximum fresh gas flow (15 l/min and continuous flush), there was still a significant pressure loss in the reservoir bag. Therefore, the pressure-limiting valve was set to 70 mbar, an oropharyngeal airway (Guedel airway) was inserted and a two-person bag-mask ventilation technique was used. Equipment failure or disconnection within the ventilatory circuit was excluded by a systematic check according to SOP by the attending anaesthesia nurse and the anaesthetist. There were no signs of laryngospasm and insufficient depth of anaesthesia was deemed very unlikely as this is routinely monitored with BIS™ and relaxometry during anaesthesia induction and all values were within the target range (BIS: 30–40; TOF: 0/4). However, no thoracic excursions could be observed and the pressure loss in the reservoir bag continued, now resulting in a reduction in oxygen saturation. At a SpO_2_ of 76%, the senior physician decided to proceed to endotracheal intubation, which was performed successfully via videolaryngoscope (GlideScope^®^) on first attempt, resulting in an immediate rise in oxygen saturation. Auscultation of both lungs revealed vesicular breath sounds.

**Fig. 1:**
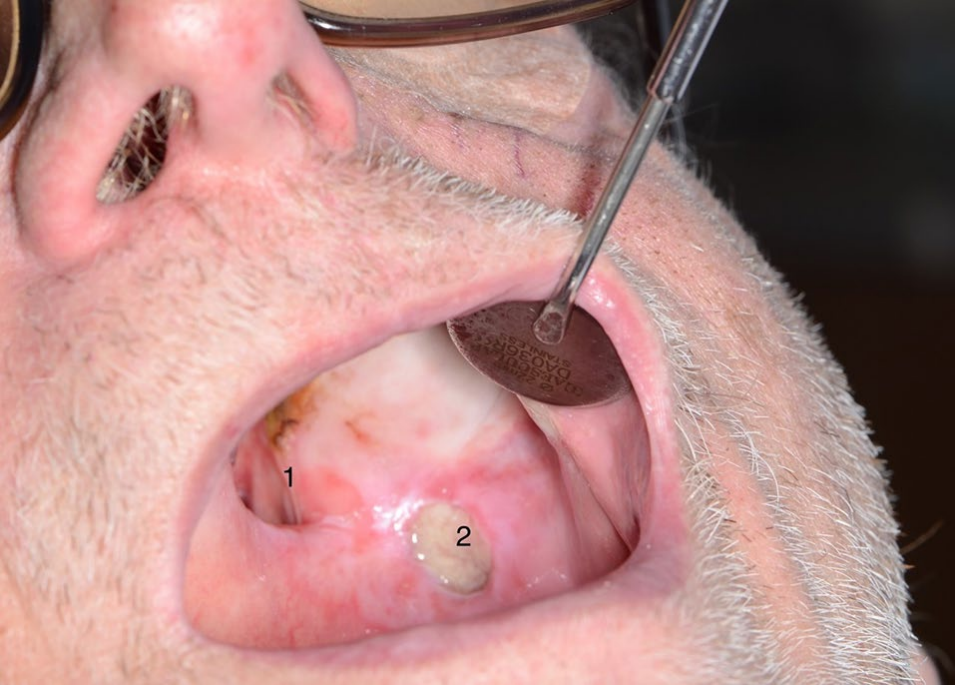
View of the fistula tract originating in the oral cavity and defect of the hard palate. (1: Fistula tract; 2: Ulcus)

**Fig. 2:**
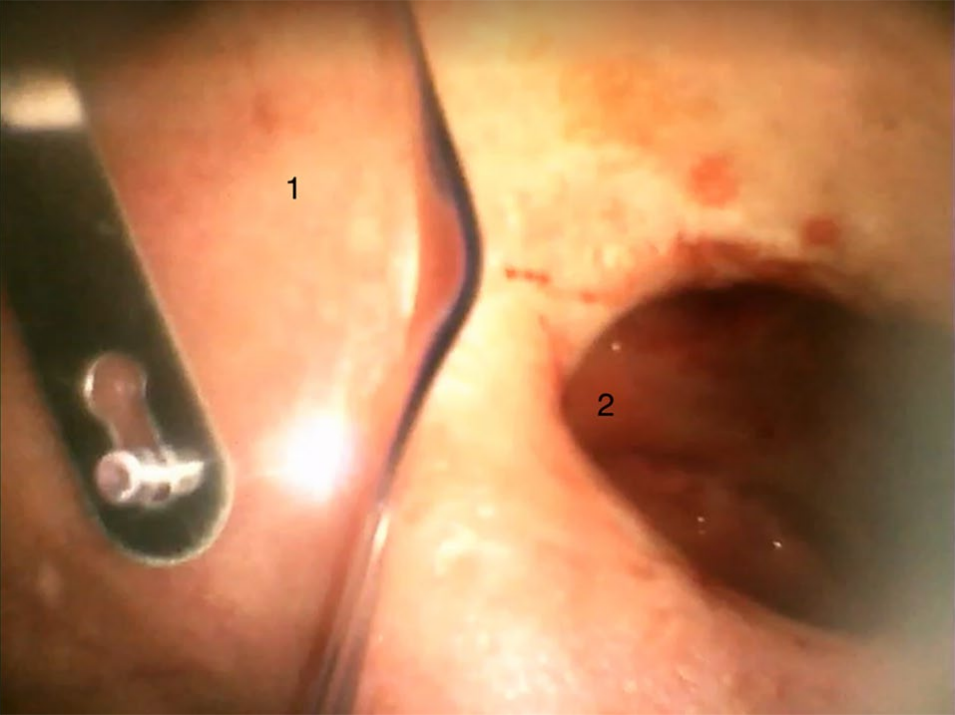
View of the eye socket (postoperative image via videolaryngoscope). (1: Oxygen mask; 2: eye socket)

**Fig. 3:**
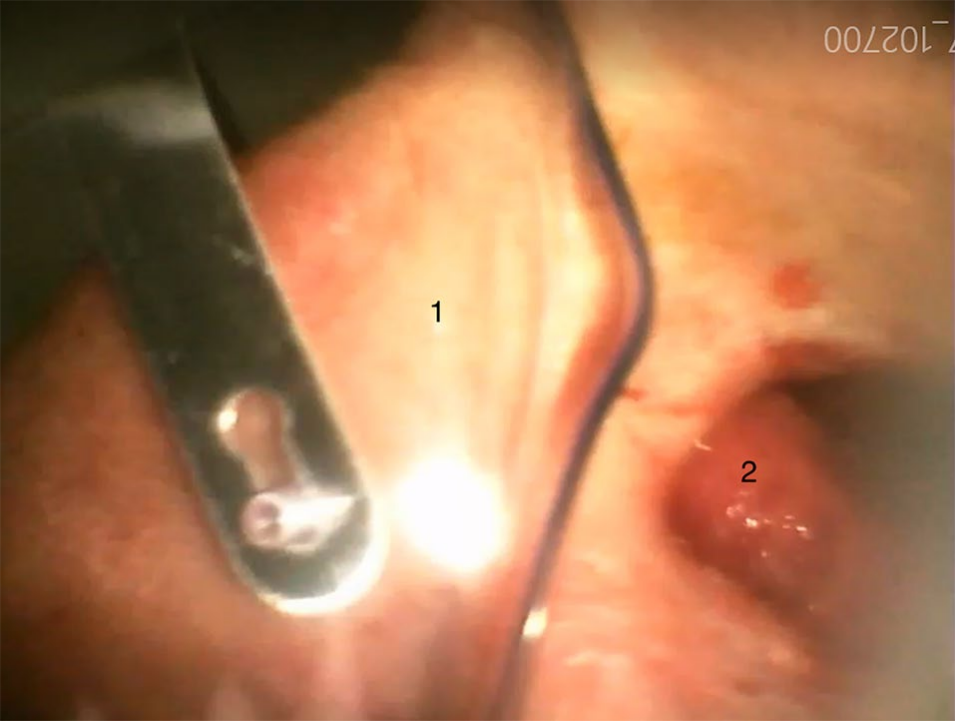
View of the eye socket with visible tongue pushing upwards through the defect in the paranasal sinuses (postoperative image via videolaryngoscope). (1: Oxygen mask; 2: tongue inside the eye socket)

The patient’s eye patch was removed to proceed with the operation and it became apparent that the left eye was missing and that there was a connection to the oral cavity. The ENT-surgeon, who entered the operating theatre directly after intubation, reported that the patient had a pronounced soft tissue and bone defect between the oral cavity and the orbit, which explained the loss of pressure during ventilation (see Figs. 1, 2 and 3). The further course of anaesthesia and surgery was uneventful and the patient recovered completely.

### Epicrisis

**Fig. 4:**
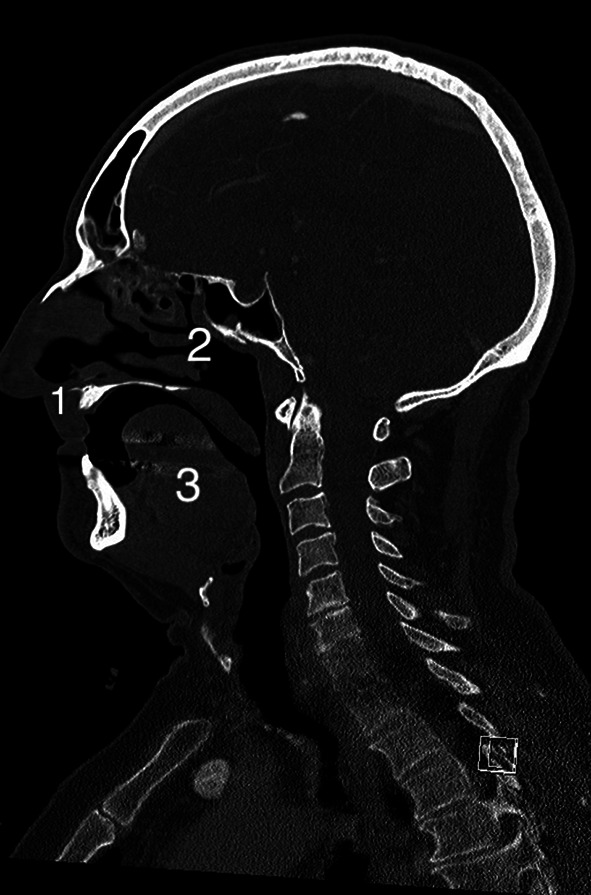
CT-scan of the head and representation of the right side of the face with recognisable anatomical structures of the nose (image taken on 11/03/2024). (1: hard palate; 2: nasal conchae; 3: tongue)

**Fig. 5:**
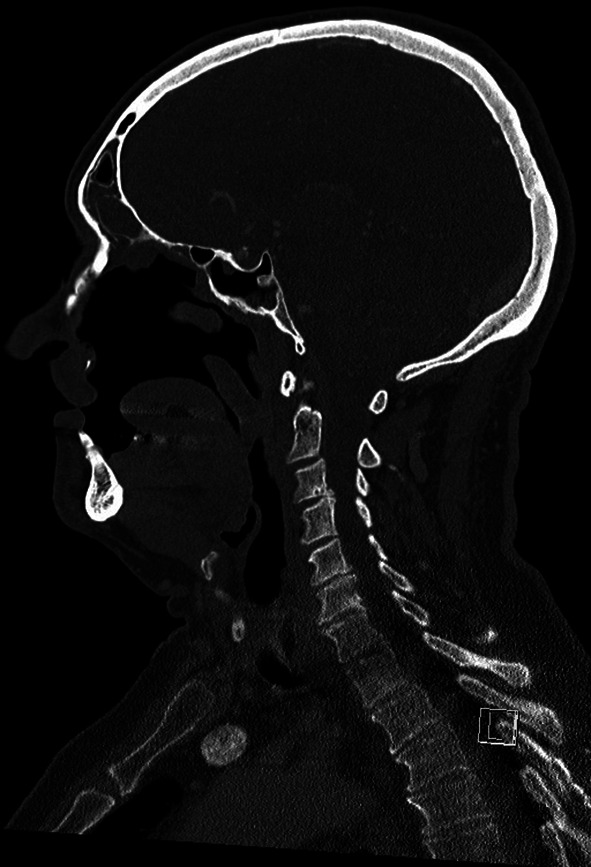
CT-scan of the head and representation of the left half of the face with missing anatomical border above the tongue (hard palate and nasal conchae) and contact with the orbit (image 11/03/2024)

During debriefing of the anaesthesia team and evaluation of the treatment records, several pieces of information were gathered that explained the unanticipated impossible facemask ventilation. In 2005, the patient underwent radical tumour surgery in the department of maxillofacial surgery for squamous cell carcinoma of the left maxillary sinus (UICC: G2, pT3, pNX, pMX, L0 V0) with exenteration of the orbit. In 2008, the patient underwent iliac crest augmentation and radial forearm flap reconstruction surgery. The following year the patient received implants to anchor the nasal epithesis. In March 2024, the patient was seen in the maxillofacial surgery department due to a new ulcer in the left maxillary sinus. Due to the inflammation, a fistula tract had developed between the oral cavity and the orbit. This resulted in an increasing defect of the bony and soft structures with partial loss of the hard palate, the paranasal sinuses and the orbital floor (see Figs. 4 and 5).

During the anaesthetic consultation, the patient did not provide any information on this part of his medical history, only mentioning a dental operation. The anaesthetist was therefore unaware of the tumor and the associated surgery. If patients do not make conspicuous statements in their self-report, there is no need for more detailed questioning in routine, low-risk procedures. As the report from the ENT department only referred to a sinus operation, and the schedule of the premedication outpatient clinic was very tight, no further investigations were initiated, and no doubts were raised about the patient’s medical history. The denture in the upper jaw covered the fistula tract during the oral cavity examination and the epithesis worn during the visit to the anaesthetic consultation covered the orbital wound cavity, so these issues were not detected. In retrospect, it is not possible to determine with certainty why the patient did not provide the relevant information in the self-disclosure form. In most cases, patients are unaware of the relevance of the information or simply forget that they have a medical condition because they have no limitations in their daily lives.

The patient’s eye patch was not removed before induction of anaesthesia, so the connection to the oral cavity was not detected. The reasons why the eye patch was not removed before induction of anaesthesia cannot be assessed in retrospect. No unusual flow noise was heard during mask ventilation as it was probably concealed by ambient noise (instrument preparation, oxygen flow, etc.).

## Discussion

It is well known in discussions about patient safety that mistakes are always caused by a number of factors that ultimately lead to a fatal situation [2]. In this case, we can also identify some weaknesses that led to the difficult ventilation situation. Firstly, the patient did not give an adequate medical history and there was a lack of information from the ENT department about the need for surgery. In addition to specifying the procedure, it is important to state why the operation is necessary, as this will help the anaesthetist to identify any other risk factors. Time pressure in the preoperative anaesthetic consultation can also be a contributing factor, as relevant questions may not have been asked. On the day of surgery, the surgeon who knew the relevant information about the patient was not in the operating theatre when the patient received the induction of anaesthesia. In addition, the eye patch was not questioned by the attending anaesthetist, although no information about this was recorded in the anaesthetic record.

The ASA Practice Guidelines for Management of the Difficult Airway define difficult facemask ventilation as follows: “It is not possible to provide adequate ventilation (e.g., confirmed by end-tidal carbon dioxide detection), because of one or more of the following problems: inadequate mask seal, excessive gas leak, or excessive resistance to the ingress or egress of gas [3]. In general, difficult mask ventilation is rare, between 0.01 and 0.5% of cases [4]. Risk factors for difficult mask ventilation in the general population include: age > 55; body mass index > 26kg/m^2^; beard; lack of teeth; and history of snoring [5]. The risk of difficult mask ventilation and difficult intubation increases significantly in patients with head and neck pathologies, especially when patients have received radiotherapy [6, 7]. To date, there have been a few case reports of difficult mask ventilation due to fistula tracts in the head region. However, in contrast to our case, in the other published case reports the fistula tract was known prior to induction of anaesthesia [8–10].

We present a case of unanticipated impossible facemask ventilation due to a fistula between the oral cavity and the orbit, resulting in an excessive gas leak. The Difficult Airway Society (UK) recommends giving 100% oxygen and calling for help in case of difficult facemask ventilation. Afterwards, in a first step one should optimize the head position, perform head tilt-chin lift (HTCL) and jaw-thrust maneuvers, use the two-person bag-mask technique, check the equipment (circuit, mask size and form, connectors) and consider deepening the level of anaesthesia. In a second step, possible further reasons for difficult facemask ventilation should be addressed (laryngospasm, gastric distention…) and an oropharyngeal airway should be inserted (or a tracheal tube, if a relaxant has been given) [11]. These recommendations were adhered to in the case presented and, therefore, despite a lack of information from the pre-anaesthesia visit, no harm for the patient occurred.

## Conclusions for practice

- Ventilation problems can sometimes be caused by a very unusual mechanism.
- Cosmetic aids such as epitheses, eye patches, wigs, etc. should be removed for a thorough examination of the patient.
- In an ideal medical world, a brief discussion with the surgeon should take place before anaesthesia is induced to talk over anaesthesia-related key points.
- ‘If you can’t ventilate, intubate’ - If there are problems with mask ventilation, it is better to move forward than to try to optimise the mask ventilation while vital signs deteriorate. A supraglottic airway can be a helpful tool for optimising ventilation.

## Data Availability

No datasets were generated or analysed during the current study.

## Abbreviations

ENT: Ear nose and throat specialty
ASA: American Society of Anesthesiologists
WHO: World Health Organization
APL-Valve: adjustable pressure-limiting valve
SOP: Standard operating procedure
BIS: Bispectral Index
TOF: Train of Four
UICC: Union Internationale Contre le Cancer
HTCL: Head tilt-chin lift
